# Bacterial communities in paddy soil and ditch sediment under rice-crab co-culture system

**DOI:** 10.1186/s13568-021-01323-4

**Published:** 2021-12-06

**Authors:** Xu Jiang, Hui Ma, Qing-lei Zhao, Jun Yang, Cai-yun Xin, Bocong Chen

**Affiliations:** Institute of Wetland Agriculture and Ecology, Shandong Academy of Agricultural Sciences, Jinan, 250100 China

**Keywords:** Rice-crab co-culture, Paddy soil, Ditch sediment, Bacterial community

## Abstract

As an important form of sustainable agriculture, rice-crab (*Eriocheir sinensis*) co-culture is rapid developing worldwide. However, the knowledge on the bacterial communities of the different components of the system is limited. In this study, we investigated the bacterial community structure in paddy soil and ditch sediment by using high-throughput sequencing technology. The results showed that compared with the ditch sediment, the content of NH_4_^+^-N in paddy soil decreased by 62.31%, and the content of AP (available phosphorus) increased by 172.02% (*P* < 0.05). The most abundant phyla in paddy soil and ditch sediment were *Proteobacteria*, *Bacteroidetes* and *Chloroflexi*, whose relative abundance was above 65%. Among the dominant genera, the relative abundance of an uncultured bacterium genus of *Saprospiraceae* and an uncultured bacterium genus of *Lentimicrobiaceae* in paddy soil was significantly lower than ditch sediment (*P* < 0.05). Alpha diversity indicated that the bacterial diversity of paddy soil and ditch sediment was similar. The bacterial community structure was affected by the relative abundance of bacteria, not the species of bacteria. Redundancy analysis (RDA) showed that the bacterial communities in paddy soil and ditch sediment were correlated with physicochemical properties. Our findings showed that the bacterial community structure was distinct in paddy soil and ditch sediment under rice-crab co-culture probably due to their different management patterns. These results can provide theoretical support for improving rice-crab co-culture technology.

## Introduction

Rice-fish co-culture integrates rice farming and aquaculture, which cultures animals (e.g., fish, crayfish, and crab) in a paddy field during the rice planting time. Due to the extra production of aquaculture, farmers’ income has increased and farmers have become more receptive to the rice-fish co-culture system (Smajgl et al. 2015; Xie et al. 2011; Zhang et al. 2016). The fish reduced the occurrence of insect pests and weeds, which also fertilized the paddy field (Zheng et al. 2017; Zhang et al. 2010). The introduction of aquatic animals improved the utilization of resources in the co-culture system (Liu et al. 2019b). Given the negative effects on fish, pesticides and fertilizers were highly reduced in co-culture farming (Clasen et al. 2018).

The rice-fish co-culture system composes of two parts: paddy and ditch. As the main part of the system, paddy has been subjected to multiple studies on soil nutrients status, bacterial community structure, and greenhouse gas (Bashir et al. 2021; Lin et al. 2020; Li et al. 2019). The ditch is similar to traditional pond culture and plays an important role in the growth of aquaculture animal. Due to the depth of the ditch, the temperature difference between upper and lower reached 7.6 ℃ (Wang et al. 2011). The water quality index of paddy field and ditch were distinct under the rice-crayfish (*Procambarus clarkii*) system (Yu et al. 2018). Zhao et al. (2017) found that the biodiversity of the paddy field was higher than ditch sediment in the rice-fish (*Odontobutis obscurus*) co-culture system. However, no taxa was observed with significance between the paddy and ditch no matter in the 1st or 5th year of rice–fish (*Monopterus albus* and *Misgurnus* spp.) field (Zhao et al. 2021). Due to the different co-culture aquaculture animals, the differences between paddy field and ditch in different rice-fish co-culture system are specific.

Bacteria, as the most abundant and diversified population of microorganisms (Acosta-Martínez et al. 2008), plays an important role in the decomposition of organic matter, element mineralization and nutrient circulation (Edwards et al. 2015). With the rapid development of high-throughput sequencing technology, more attention has been paid to the bacterial communities in different habitats (Zecchin et al. 2017). Given the important role of bacteria in improving the environment and growth of aquatic animals, it is very important to understand the bacterial community structure of different components in the co-culture system.

The rice-crab co-culture system is a green and efficient agricultural development mode as an important form of rice-fish co-culture system. The culturing of crab in rice fields improved the quality of crab (Wu et al. 2020) and enhanced soil fertility (Hu et al. 2020). The effects of rice-crab co-culture on bacterial community structure and diversity in paddy fields have been reported (Cheng et al. 2017), but the study about ditch is relatively limited. Given the specific of different rice-fish co-culture system and the distinct between water quality parameters of paddy field and ditch under the rice-crab co-culture system (Zhang et al. 2013), we speculate that there are more significant differences between the paddy and ditch under the rice-crab co-culture system. The main purpose of our present study was to clarify the physicochemical properties and bacterial community structure characteristics of paddy soil and ditch sediment and to reveal the differences between them under the rice-crab co-culture system. The current study can provide theoretical support for the development of rice-crab co-culture technology.

## Materials and methods

### Study area

The experiment was conducted in Dongying City, Shandong Province, China (37° 40′ 43″ N, 118° 54′ 12″ E). This area has a warm temperate continental monsoon climate where rice cropping is the major farming system. TN (total nitrogen), TP (total phosphorus), AN (available nitrogen) and AP (available phosphorus) in paddy soil were 0.78 g kg^−1^, 0.68 g kg^−1^, 59.19 mg kg^−1^ and 5.38 mg kg^−1^, respectively.

### Experimental design

To investigate all parameters of paddy soil and ditch sediment, three replicates were represented by a 900 m^2^ plot. Each plot had an independent water inlet, outlet and a U-type ditch (upper width 1.5 m, lower width 0.8 m, depth 1.0 m). The initial physicochemical properties of paddy soil and ditch sediment were the same as shown above.

The rice variety of “Shengdao 19” was sown on June 5 with direct sowing, and the seed amount of rice was 150 kg ha^−1^. Fertilizer for rice (N:P:K fertilizer, 15:15:15, 525 kg ha^−1^) was broadcast to the surface of the experimental paddy field. No pesticides or herbicides were used during the experiment.

The crabs (each 15 g) were released into each plot on June 20, 2020. The stocking density was 4500 individuals ha^−1^. The crabs were fed 3% ~ 5% of the total crab weight daily with pellet feed, and the daily food consumption was adjusted according to the feeding conditions of the crabs and the weather conditions.

### Sample collection

Three soil samples were randomly collected from the paddy field of each plot, and three sediment samples were collected from the middle of the three sides of the ditch on October 30, 2020. After the samples were fully mixed, they were divided into two parts that were used for (i) determination of soil physicochemical properties, (ii) determination of bacterial community.

### Determination method

TN was determined by the Kjeldahl method with sulfuric acid + catalyst digestion. TP was analyzed by the sodium hydroxide melt-molybdenum blue colorimetry. AN content was identified by the alkali-hydrolytic diffusion method. The content of AP was determined by the Olsen method. NH_4_^+^-N (ammonia nitrogen) content was determined by using the kit of Suzhou Keming Biotechnology limited company.

### Microbiome analysis

For microbiome analysis, three replicates were used for each group. Total genomic DNA was extracted from soil and sediment samples using FastDNA Soil Kit (MP Biomedicals, USA) following the manufacture's protocol. The High-throughput sequencing of the samples was conducted by the Beijing Biometrics limited company. 16S full-length region primer: 27F_(16S-F): AGRGTTTGATYNTGGCTCAG; 16 s–492 r_ (R): TASGGHTACCTTGTTASGACTT.

### Data statistics and analysis

After sequences were identified and chimeras were removed, Usearch software was used to cluster reads at 97.0% similarity level to obtain OTU. Qiime2 software was used to evaluate the Alpha diversity index of the samples, and Canoco 5.0 software was used to complete RDA analysis. The differences in physicochemical properties, bacterial diversity index and relative abundance of the samples were analyzed by SPSS 21.0 t-test at *P* < 0.05.

## Results

### Physicochemical properties of paddy soil and ditch sediment

As shown in Table 1, the content of TN, TP, AN and AP in paddy soil were higher than those in ditch sediment, especially the content of AP was significantly increased by 172.02% (*P* < 0.05). The content of NH_4_^+^-N was significantly decreased by 62.31% (*P* < 0.05) compared with ditch sediment.

**Table 1:** Physicochemical properties of paddy soil and ditch sediment

	TN (g kg^−1^)	TP (g kg^−1^)	AN (mg kg^−1^)	AP (mg kg^−1^)	NH_4_^+^-N (mg kg^−1^)
B	0.81 ± 0.20	0.73 ± 0.04	62.40 ± 15.55	7.29 ± 2.82^a^	8.49 ± 1.41^a^
S	1.25 ± 0.40	0.83 ± 0.16	82.13 ± 15.62	19.83 ± 2.15^b^	3.20 ± 0.68^b^

B: ditch sediment; S: paddy soil. Values in the same column with different letters are significantly different (*P* < 0.05). The same as below

### Overview of sequencing results

**Fig. 1:**
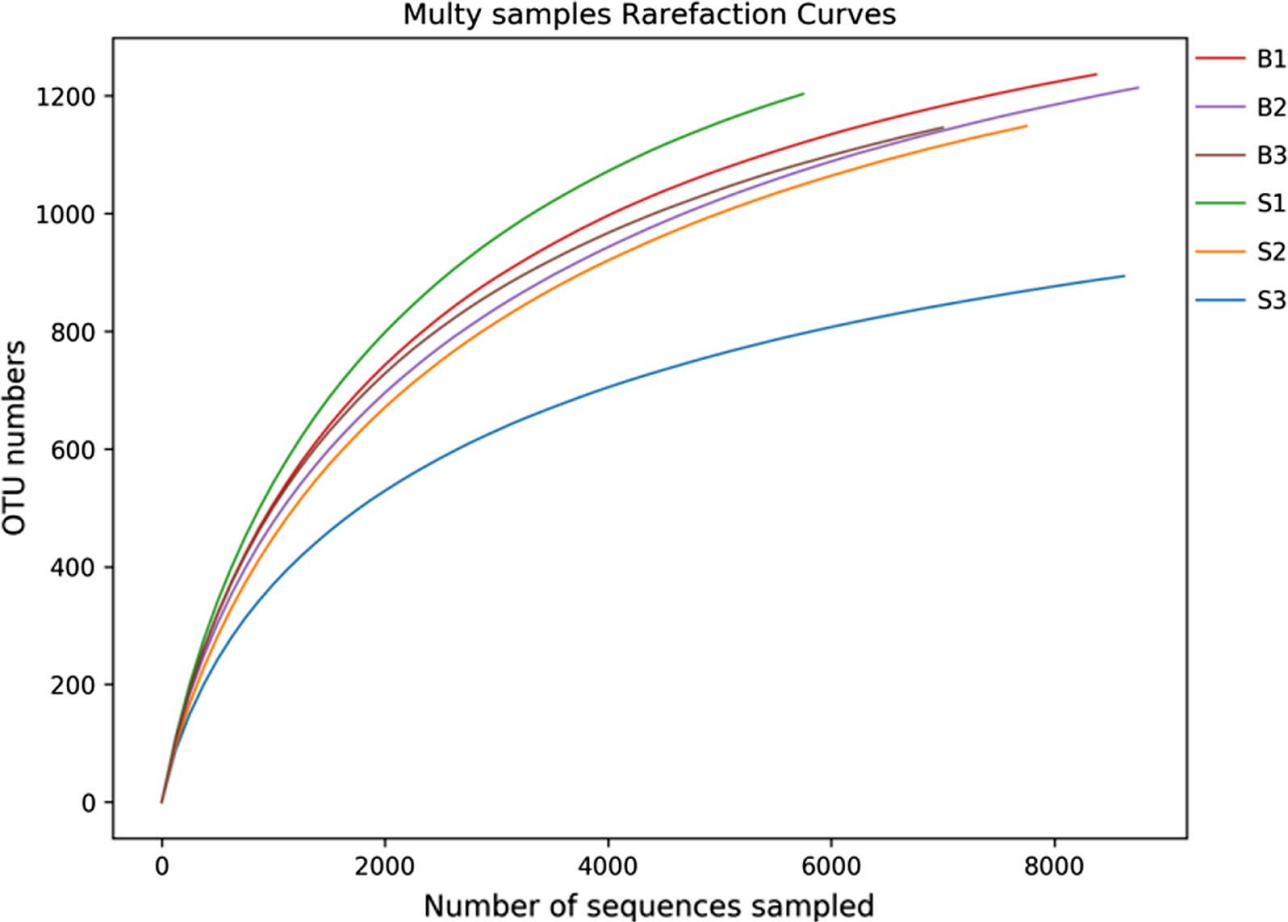
Rarefaction curve of different samples

A total of 75,024 CCS sequences were obtained from 6 samples. Each sample generated 9913 CCS sequences at least, with an average of 12,504 CCS sequences and an average length of 1456 bp. As shown in Fig. 1, the rarefaction curves gradually flattened as the number of sample sequences increased, which was indicating that data obtained by sequencing covered most of the bacterial species from the samples.

### Bacterial diversity analysis

**Fig. 2:**
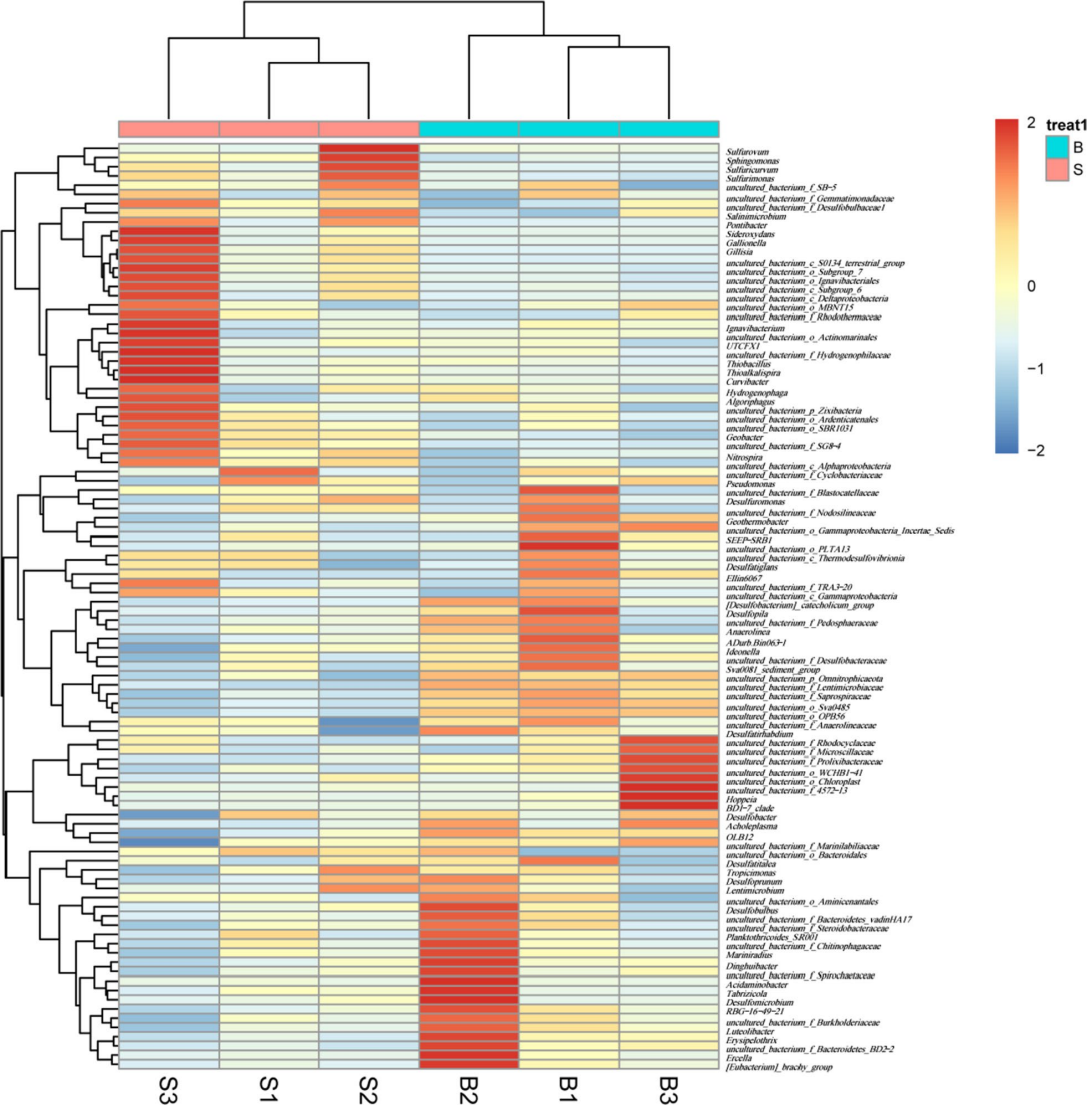
Bacterial community heatmap analysis in genus level

Alpha diversity reflects the species abundance and diversity of the sample. The species abundance was measured by Chao1 and Ace indices. The number of species and diversity was measured by the Shannon and Simpson indices. As shown in Table 2, ACE, Chao1, Simpson and Shannon indexes of ditch sediment were 8.36%, 10.77%, 1.01% and 5.05% higher than those of paddy soil. But, there was no significant difference among them (*P* > 0.05), which indicated that the bacterial diversity was similar between the ditch sediment and paddy soil. The community heatmap at the genus level showed that all samples clustered into two clusters of paddy soil and ditch sediment (Fig. 2).

**Table 2:** Alpha diversity index of paddy soil and ditch sediment

	ACE	Chao1	Simpson	Shannon
B	1418.89 ± 47.08	1432.19 ± 31.84	1.00 ± 0.00	9.16 ± 0.10
S	1309.48 ± 208.00	1292.99 ± 183.70	0.99 ± 0.00	8.72 ± 0.67

### Analysis of bacterial community composition

**Fig. 3:**
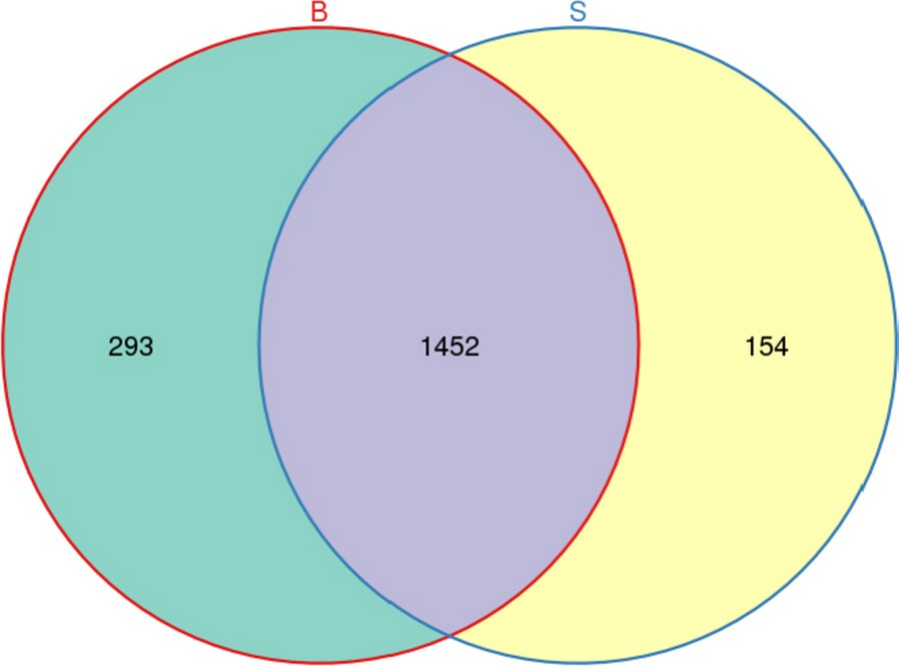
The Venn diagram of different samples at OTU level

1899 OTUs were obtained by clustering at 97.0% similarity level. Venn diagram (Fig. 3) demonstrated that 293 and 154 OTUS only appeared in the ditch sediment and paddy soil. 1452 OTUs were found in both paddy soil and ditch sediment, accounting for 76.46%.

**Fig. 4:**
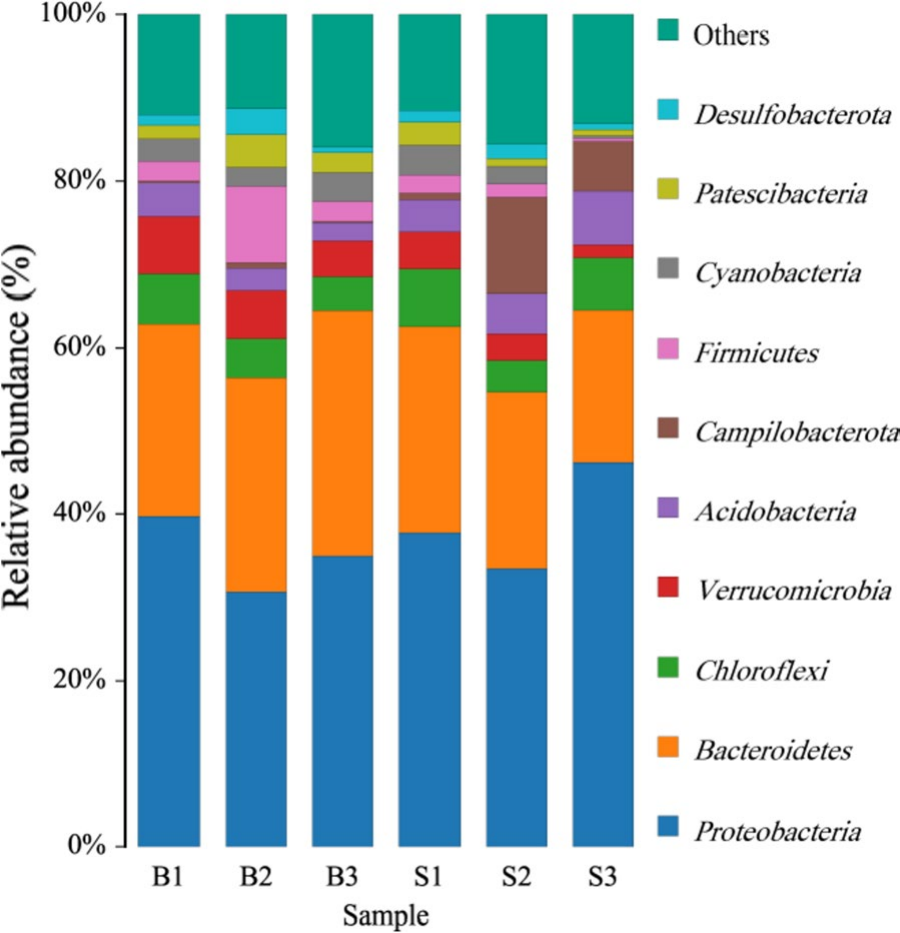
Percentages of the dominant phyla in paddy soil and ditch sediment

**Fig. 5:**
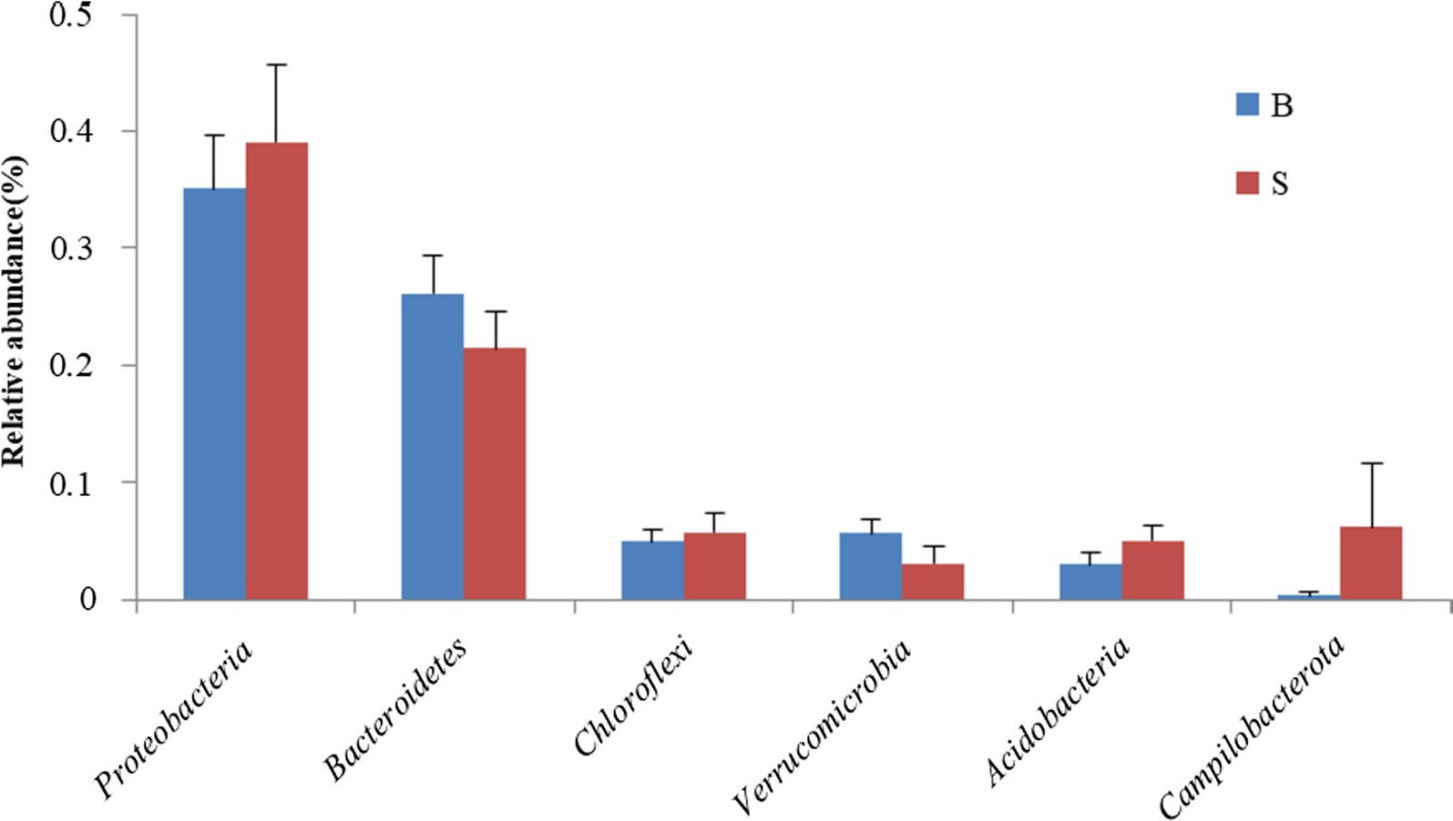
The differences with the significance of the top 6 phyla in paddy soil and ditch sediment

The 1899 OTUs were annotated into 50 phyla, 119 classes and 615 genera. Figure 4 demonstrated the relative abundance of the dominant phyla in paddy soil and ditch sediment. The dominant phyla was defined with 5% as the dividing line. *Proteobacteria*, *Bacteroidetes*, *Chloroflexi*, *Campilobacterota* and *Acidobacteria* were the dominant phyla in paddy soil, accounting for about 78% of the total sequence. *Proteobacteria*, *Bacteroidetes*, *Chloroflexi* and *Verrucomicrobia* were the dominant phyla in the ditch sediments, accounting for about 72% of the total sequence. Based on the t-test, the relative abundance of dominant phyla in paddy soil and ditch sediment did not reach a significant difference level (Fig. 5, *P* > 0.05).

**Fig. 6:**
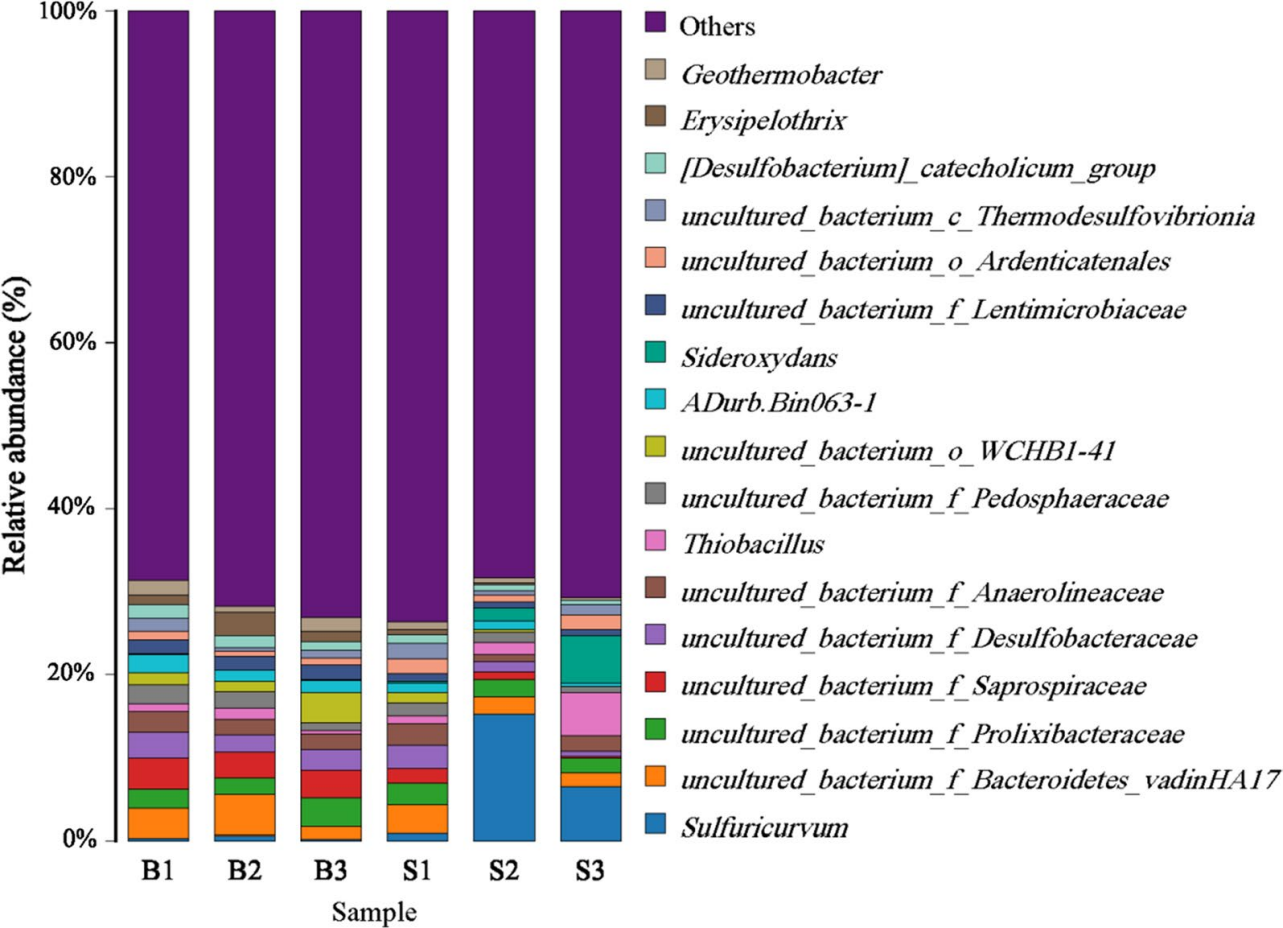
Percentages of the dominant genera in paddy soil and ditch sediment

**Fig. 7:**
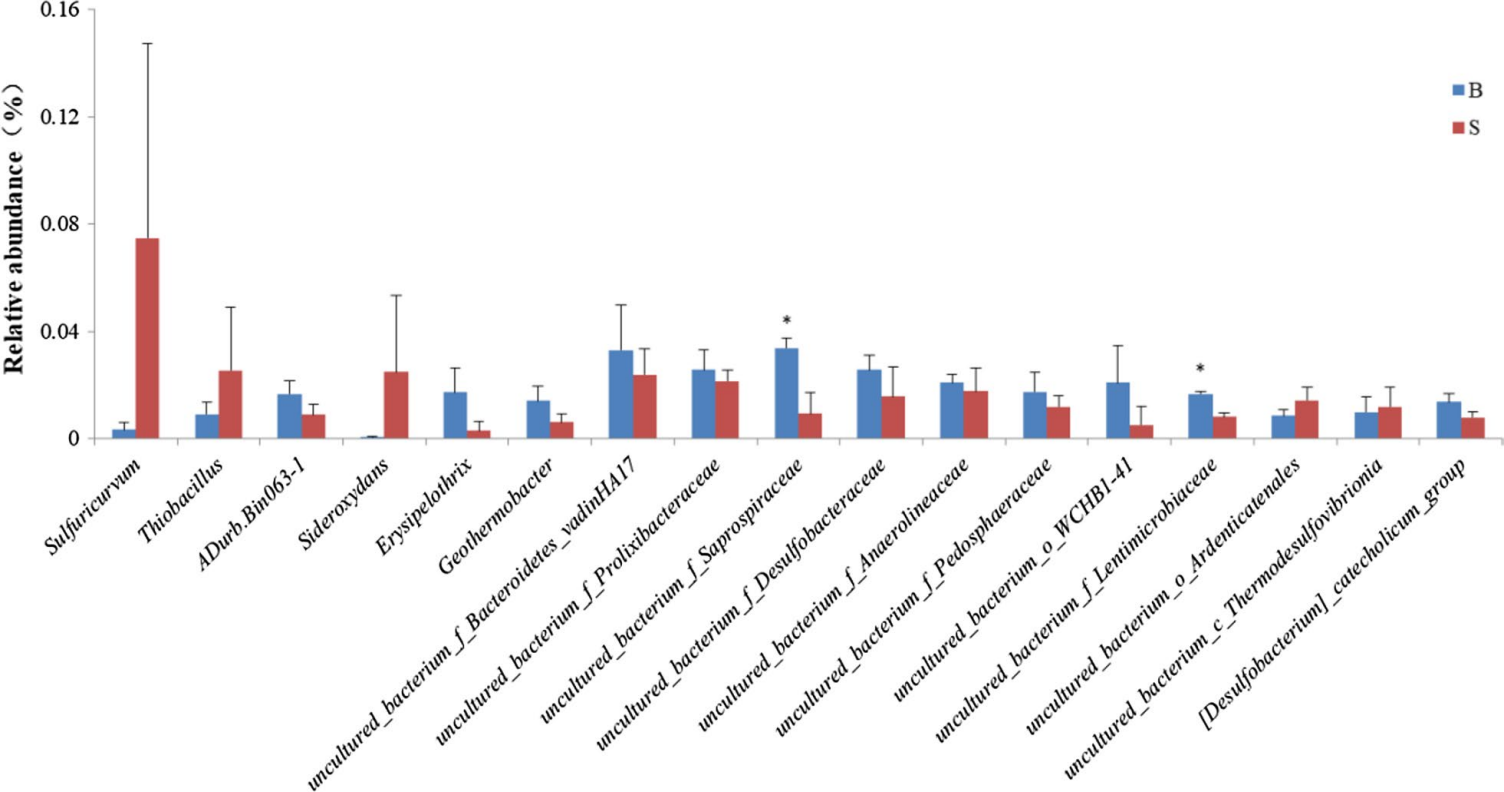
The differences with the significance of the top 17 genera in paddy soil and ditch sediment (* indicates *P* < 0.05)

The dominant genera with abundance > 1% were selected to make a histogram of relative abundance (Fig. 6). About 71% of the samples were from other genera (relative abundance < 1%). There were 10 species and 12 species of dominant genera in paddy soil and ditch sediment, respectively. Among them, there were 5 common dominant genera: an uncultured bacterium genus of *Bacteroidetes_Vadinha17*, an uncultured bacterium genus of *Desulfobacteraceae*, an uncultured bacterium genus of *Anaerolineaceae*, an uncultured bacterium genus of *Prolixibacteraceae* and an uncultured bacterium genus of *Pedosphaeraceae*. Among the dominant genera, the relative abundance of an uncultured bacterium genus of *Saprospiraceae* and an uncultured bacterium genus of *Lentimicrobiaceae* in paddy soil was significantly lower than ditch sediment (*P* < 0.05), while the relative abundance of other genera was not significantly different (Fig. 7, *P* > 0.05).

### Influencing factors of bacterial community

**Fig. 8:**
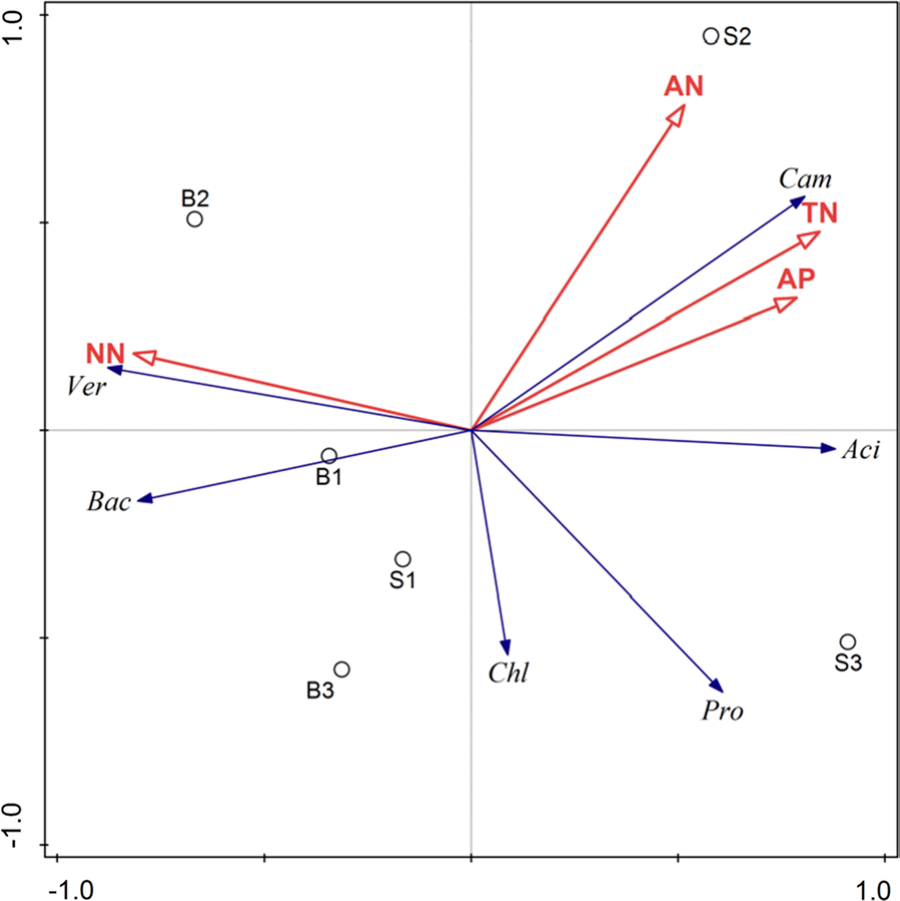
RDA of 6 dominant phyla and environmental factors. Pro: *Proteobacteria*, Aci: *Acidobacteria*, Cam: *Campilobacterota*, Bac: *Bacteroidetes*, Ver: *Verrucomicrobia*, Chl: *Chloroflexi*

Redundancy analysis (RDA) was carried out for dominant phyla and physicochemical properties in paddy soil and ditch sediment under the rice-crab co-culture system. TP was eliminated because of the absence of significant explanations. The results showed that the explanatory rate of environmental factors was 81.46% at the phyla level. As shown in Fig. 8, NH_4_^+^-N had a significant positive correlation with *Bacteroidetes* and *Verrucomicrobia*, and a significant negative correlation with *Proteobacteria*, *Acidobacteria* and *Campilobacterota*. TN, AP and AN had a significant positive correlation with *Acidobacterota* and *Campilobacterota*, and a significant negative correlation with *Bacteroidetes* and *Verrucomicrobia*.

## Discussion

Fertilizer improves soil fertility significantly, and the continuous input of feed also increases nutrient elements in the sediment (Liang et al. 2013; Liu et al. 2019a). Our study found that TN, TP, AP and AN in paddy soil and ditch sediment increased compared with the initial stage under the rice-crab co-culture system. The physicochemical properties of paddy soil increased by 60.26%, 22.06%, 138.76% and 268.59%, respectively. Among them, the available nutrients increased greatly. However, the physicochemical properties of the ditch sediment changed little. AP increased by 35.50%, while the contents of TN, TP and AN increased by only 3.85–7.35%. A large amount of feed input resultes in a significant increase in the residual feed, which leads to the problem of eutrophication in the traditional high-density pond culture (Youssouf et al. 2012). However, both the density of the crab and the amount of feed were reduced in the rice-crab co-culture system. Meanwhile, the crab excrement was released in the paddy field and used by rice, so minor changes have taken place in the physicochemical properties of the ditch sediment. Consistent with our findings, Lv et al. (2014) reported that a small amount of feed would not change the N and P contents of sediment. The distribution of N and P elements in the system was not uniform despite the effects of diffusion, migration, crab movement and metabolism. The AP in paddy soil was significantly higher than ditch sediment, while the NH_4_^+^-N content was the opposite. Significant differences in the physicochemical properties between paddy soil and ditch sediment have taken place. Yang et al. (2020) reported that the soil fertility index in the ditch of the rice-shrimp (*Macrobranchium nipponense*) co-culture system was lower than paddy soil, which was similar to the results in this study. NH_4_^+^-N may be affected by different water management methods. Paddy alternated wet and dry for part-time, while ditch was permanently flooded. The alternation of wet and dry will enhance soil nitrification potential, thus resulting in a decrease of NH_4_^+^-N content (Kuang et al. 2019). Furthermore, biological enrichment and surface agglomeration may also have contributed to the differences. Rice absorbs nutrients from the soil, and some nutrients are returned to the surface soil of the paddy field in the form of dead branches and fallen leaves. As a result, the content of nutrients on the paddy field is higher than the ditch sediment.

*Proteobacteria*, *Bacteroidetes* and *Chloroflexi* were the common dominant phyla of the paddy soil and ditch sediment, which widely found in anaerobic sludge, farmland soil and other environments (Ahring 2003; Fierer and Jackson 2006; Janssen 2006). They also play important roles in nitrogen removal, sulfide oxidation and carbohydrate metabolism (Thomsen et al. 2007). *Acidobacteria* and *Campilobacterota* were the other dominant phyla in paddy soil. *Acidobacteria* participated in the carbon cycle of humus decomposition (Liu et al. 2014), which had a high relative abundance in the absence of water level (Zeglin et al. 2011). *Campilobacterota* had a function of oxidizing sulfide (Carrier et al. 2020). *Verrucomicrobia* has dual environmental effects of biological nitrogen fixation and mitigation of methane emission as dominant phyla in the ditch sediment (Nixon et al. 2019; Dunfield et al. 2007; Chiang et al. 2018), and its distribution level may be affected by soil moisture content (Daniel and Thomas 2001).

To enhance the identification accuracy, the sequence length was all over 1000 bp by using full-length sequencing technology. Analysis of the bacterial community showed that most of the dominant genera were uncultured. Among them, the relative abundance of an uncultured genus of *Saprospiraceae* and an uncultured genus of *Lentimicrobiaceae* in the ditch sediment was significantly higher than paddy soil, both belonging to *Bacteroidetes*. Xia et al. (2008) reported that members of the genus uncultured *Saprospiraceae* played an important role in protein degradation by the production of extracellular enzymes. Reza and Alvarez (2016) and Silva et al. (2017) revealed that unknown bacteria of *Saprospiraceae* may have been responsible for the successful nutrient removal. *Lentimicrobium* was strictly anaerobic short bacillus, which can also metabolize glucose and other small molecular organic matter (Sun et al. 2016). The difference in the relative abundance of the two genera is probably influenced by the substrate abundance. To keep the healthy growth of crab, the feed was continued to be put into the ditch, which improved carbohydrate content in the ditch sediment. The bacteria metabolizing on carbohydrates as substrate increased significantly, thus affecting the relative abundance of the bacterial. The relative abundance of *Sulfuricurvum* in paddy field soil (7.48%) was much higher than ditch sediment (0.32%), but there was no significant difference between them. The variation range of *Sulfuricurvum* in the paddy field was 0.79% ~ 15.18%. *Sulfuricurvum* was regarded as an important genus. *Sulfuricurvum* belongs to a facultative sulfur-oxidizing bacterium with chemoautotrophic capacity and can oxidize sulfur compounds with oxygen or nitrate as electron acceptor (Kodama 2004). We assumed that the relative abundance of *Sulfuricurvum* was enhanced in the wake of elevated oxygen content in paddy soil which was caused by the rice root aerenchyma and wet-drying management method. Relevant studies reported that sulfide in the sediment will be gradually oxidized with the increase of dissolved oxygen in overburdened water (De et al. 2012), which is consistent with the conclusion of our research.

There was little difference in bacterial species between paddy soil and ditch sediment, mainly manifested by changes in relative abundance based on the analysis of bacterial community composition. Zhang et al. (2019) found that both water and fertilizer measures and water control treatment could change microbial abundance, but had little influence on microbial species.

The distribution of the bacterial community was affected by environmental factors (Gu et al. 2017). The different management methods of paddy and ditch caused the change of environmental factors. Through RDA analysis, NH_4_^+^-N had a significant positive correlation with *Bacteroidetes* and *Verrucomicrobia* which were important participants in the N cycle (Zheng et al. 2009). NH_4_^+^-N affected the community structure of ammonia-oxidizing microorganisms (Verhamme et al. 2011), which is consistent with this conclusion. Arnds et al. (2010) reported that the relative abundance of *Verrucomicrobia* decreased with the elevated phosphorus content in lakes rich in humus. *Campilobacterota* and *Acidobacteria* were affected by TN and AP. Both nitrogen and phosphorus may lead to differences in the abundance and community structure of soil bacteria (Karasu and Dogan 2009; Rinklebe and Langer 2006).

In conclusion, the physicochemical properties in the paddy and ditch were differentiated under the rice-crab co-culture system, probably due to the different management methods which had little effect on the bacterial species in the two parts of the system. However, the bacterial community structure was affected by the relative abundance of bacteria, not the species of bacteria. In this study, the distinction of the physicochemical properties and bacterial community between paddy soil and ditch sediment was clarified for the first time, which is only a preliminary exploration. Further study are still necessary in the effects of bacterial community on the environment and rice and co-culture animals, thus to provide a reference for the optimization of co-culture technology.

## Data Availability

All the sequences can be downloaded from the NCBI Sequence Read Archive Database under the accession numbers PRJNA734090.
